# Reviving Forgotten Foods: From Traditional Knowledge to Innovative and Safe Mediterranean Food Design

**DOI:** 10.3390/foods15010150

**Published:** 2026-01-02

**Authors:** Manica Balant, Judit Català-Altés, Teresa Garnatje, Fuencisla Cáceres, Clara Blasco-Moreno, Anna Fernández-Arévalo, Clàudia Knudsen, Valeria De Luca, Jana Peters, Ignacio Sanz-Benito, Marc Casabosch, Marc Talavera, Esther López-Viñallonga, Carla Cárdenas Samsó, Natàlia Cuberos-Sánchez, Anabel Cepas-Gil, Joan Vallès, Airy Gras

**Affiliations:** 1 Laboratori de Botànica–Unitat Associada CSIC, Facultat de Farmàcia i Ciències de l’Alimentació, Institut de Recerca de la Biodiversitat, Universitat de Barcelona, Avinguda Joan XXIII, 27-31, 08028 Barcelona, Catalonia, Spain; juditcatalaaltes@ub.edu (J.C.-A.); fcaceres@ub.edu (F.C.); joanvalles@ub.edu (J.V.); 2 Institut Botànic de Barcelona (IBB), CSIC-CMCNB, Passeig del Migdia, s/n, Parc de Montjuïc, 08038 Barcelona, Catalonia, Spain; tgarnatje@ibb.csic.es; 3 Jardí Botànic Marimurtra–Fundació Carl Faust, Passeig de Carles Faust, 9, 17300 Blanes, Catalonia, Spain; 4 Cooperativa Eixarcolant SCCL, Carrer Major, 47, 08719 Jorba, Catalonia, Spain; 5 E.I. Sambucus SCCL, Edifici Mercat Municipal, Carrer del Pintor Guàrdia, 6, 08560 Manlleu, Catalonia, Spain; 6 Fundació Emys, Carrer Santa Coloma, Km 21.1, 17421 Riudarenes, Catalonia, Spain; 7 Xarxa per a la Conservació de la Natura (XCN), Carrer de Sant Jordi, 65, 08500 Vic, Catalonia, Spain; 8 Institut d’Estudis Catalans (IEC), Carrer del Carme, 47, 08001 Barcelona, Catalonia, Spain

**Keywords:** ethnobotany, Mediterranean forests, non-timber forest products, sustainable food systems, traditional knowledge, wild edible plants

## Abstract

Global food security and dietary diversity depend on identifying novel and sustainable food sources. Wild edible plants (WEPs) traditionally used in Mediterranean regions offer considerable potential due to their rich history of use. Here, ethnobotanical knowledge was systematically compiled for the fruits and cones of five taxa (*Arbutus unedo*, *Prunus spinosa*, *Quercus* spp., *Pinus* spp. and *Rosa* spp.), documenting alimentary uses, preparation and conservation methods across diverse food categories. Analysis of over 2800 traditional use reports identified 54 distinct alimentary uses from 16 categories, with raw consumption and sweet preserves being the most prevalent. *Rosa* spp. exhibited the highest diversity of uses (36), whereas the family Pinaceae showed the lowest (19). Statistically significant associations between individual fruits and specific food preparations were also observed, offering guidance for innovative product development. Information on processing methods that preserve nutritional components, along with documentation of potential harmful effects and the methods to mitigate them, was collected, providing essential guidance for developing safe and functional alimentary products. Together, traditional knowledge, regulatory adherence, and sustainable practices create new opportunities to develop innovative, safe, culturally grounded, and sustainable food products that enrich diets and preserve cultural and ecological heritage.

## 1. Introduction

For millennia, human activities have left a lasting imprint on the Mediterranean landscape, where forests and people have coexisted and influenced each other through cultivation, management, and use [1]. However, recent decades have seen significant shifts in land use, rural depopulation, and abandonment of traditional agricultural practices, resulting in altered forest structure and dynamics across the Mediterranean basin [2,3,4,5]. Although forest cover in the region has been increasing [3,6], high extraction costs and low profitability have limited proper forest management [6,7], leading to fuel accumulation and increased vulnerability to large wildfires [8,9]. Within this context, promoting non-timber forest products (NTFPs), such as plant resins, fruits, nuts, cork, mushrooms, and medicinal, edible and aromatic plants, offers a promising mechanism to reactivate forest-based economies [10]. NTFPs can provide additional income that can contribute to sustainable forest management, which improves ecosystem resilience, enhances biodiversity, and supports rural economies [11,12].

Among NTFPs, wild edible plants (WEPs) especially stand out. They offer a sustainable resource that can complement conventional agriculture, particularly in the Mediterranean region, which is increasingly affected by water scarcity [9]. These plants grow spontaneously, are well adapted to local conditions, and require minimal irrigation or chemical input [13,14]. Integrating WEPs into land management therefore reduces pressure on water-demanding crops, preserves local ecosystems, and enhances biodiversity [13,15]. Because many Mediterranean WEPs used for food are abundant and non-endangered [16], sustainable harvesting is feasible when properly regulated. By integrating harvesting of WEPs into forest management plans, landowners could be incentivized to maintain and enhance populations of fruit-bearing native species, improving forest structure, biodiversity, and fire resilience [13,17,18,19].

Yet, the potential of WEPs remains largely unrealized due to the progressive erosion of traditional ecological and ethnobotanical knowledge. Across Europe, traditional knowledge has progressively declined due to negative social stigma associated with use of WEPs, massive rural-to-urban migration, and socio-economic transformation [4,5,20]. The negative stigma, together with geographic and cultural disconnection, has disrupted the vertical transmission of knowledge from generation to generation, resulting in the loss of plant-related practices and a drastic decline in the gathering and consumption of wild plant species [21,22].

The consequences extend beyond cultural heritage. Today, only 30 crop plant species provide 90% of global caloric intake, with more than four billion people relying on rice, maize and wheat [23]. This highlights a critical loss of food diversity and increased vulnerability of food systems. By 2050, the global population is expected to have increased tenfold since 1800, intensifying the need for new and sustainable food sources [23]. In this context, FAO recommendations stress the importance of crop wild relatives and WEPs for strengthening the resilience and sustainability of food systems [24]. The use of WEPs broadens dietary diversity, diversifies sources of food, and supports ecosystem biodiversity. Additionally, WEPs can enhance the flexibility of food systems and improve access to fresh, nutritious foods during crises by reducing dependence on distant supply chains, uniform, high-risk production systems, and reinforcing locally oriented markets [25].

Traditional knowledge preserves valuable information not only regarding the diversity of available WEPs, but also their processing methods [26]. It encompasses warnings about toxicity, adverse effects, and preparation methods designed to mitigate such risks [27,28]. This knowledge therefore represents a valuable resource for the development of new, safe, and innovative food products [20,29]. WEPs have recently attracted growing public interest, reflected in publication of field guides, organization of foraging and wild food workshops [30], their inclusion in restaurant menus [13,31], and commercialization by companies [32,33]. Additionally, they are increasingly recognized for their value as cultural ecosystem services [34]. Nevertheless, their economic potential remains largely untapped despite the growing demand for sustainably sourced, highly nutritious foods [7,13,30]. Reviving traditional knowledge of WEPs is therefore essential—not only to safeguard cultural heritage, but also to diversify local food systems, enhance food security and sovereignty, and contribute to sustainable agricultural and forest management [35,36,37,38,39].

**Figure 1:**
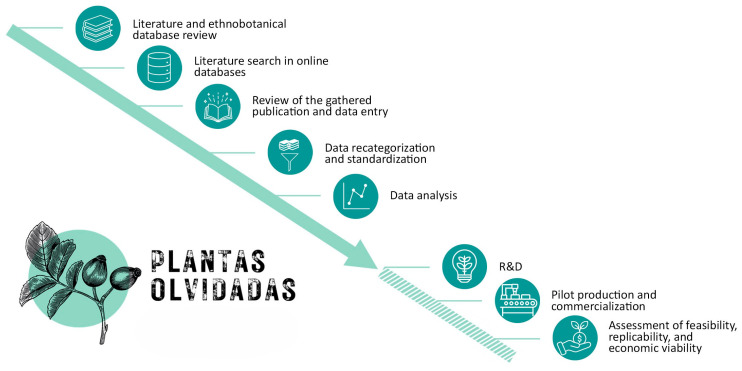
Schematic representation of the *Plantas olvidadas* project workflow, beginning with a literature review and continuing with the data recategorization and analysis (shown as a solid green arrow). The results obtained will serve as inspiration for the research and development process (R&D) of innovative products, followed by product commercialization and the assessment of feasibility, replicability, and economic viability to be carried out afterward (shown with an interrupted green pattern).

In light of these considerations, a two-year interdisciplinary project *Plantas olvidadas* (Forgotten Plants) was launched in 2024 in Catalonia (NE Iberian Peninsula, Western Mediterranean) [40]. One of the main objectives of the project is to integrate traditional knowledge with the development of innovative, locally produced food products that support alternative forest-based economies while advancing sustainable forest management. The project focuses on five taxa (*Arbutus unedo* L., *Prunus spinosa* L., *Quercus* spp., *Pinus* spp. and *Rosa* spp.), characteristic of Mediterranean forest and forest-edge habitats and notable for their abundance [41,42,43,44,45,46,47,48], long history of alimentary use [26,49,50,51,52,53,54,55], and diverse nutritional profiles [13,41,56,57,58,59,60,61,62,63,64,65,66,67,68], demonstrating their economic potential as functional foods. The insights from traditional knowledge will provide a foundation for the next phase of the project, which will focus on the research and development of innovative, safe, and functional alimentary products (Figure 1). A selection of these products will be produced on a pilot scale and introduced to the market, followed by an evaluation of feasibility, replicability, and economic viability, to assess the effectiveness of the approach (Figure 1). Although the experimental part of the project is currently limited to Catalonia, it aims to expand into other forested areas of the Mediterranean, promoting replicable multifunctional forest management approaches and associated ecological, social, and economic benefits across the region.

Although these five taxa possess a long and well-documented history of use and recognized nutritional value, they remain underutilized in contemporary food systems. This study focuses on the first objective of the *Plantas olvidadas* project: to identify effective ways of applying traditional alimentary knowledge to the development of innovative and safe food products derived from the five target taxa. Here, we conducted a comprehensive review of existing traditional knowledge of alimentary use and analyzed the resulting dataset to support the subsequent development of food products inspired by these traditional uses.

## 2. Materials and Methods

### 2.1. Overview of Selected Taxa

**Figure 2:**
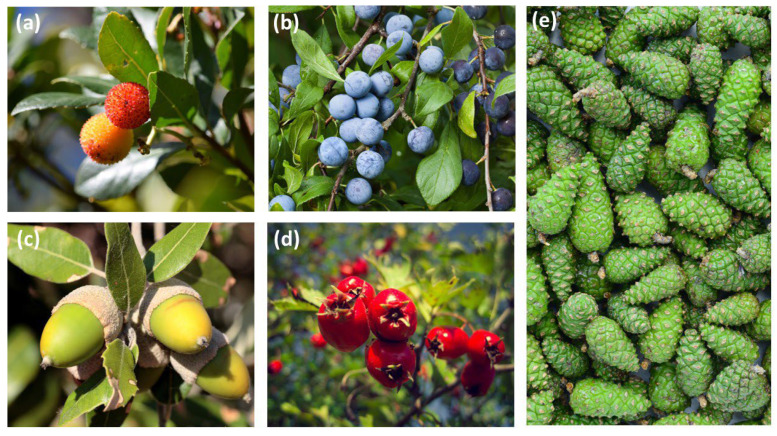
The five taxa considered here: (**a**) *Arbutus unedo*, (**b**) *Prunus spinosa*, (**c**) *Quercus* spp., (**d**) *Rosa* spp., and (**e**) *Pinus* spp.

*Arbutus unedo* L. (strawberry tree) is a small evergreen shrub or tree, native to the Mediterranean basin and Asia Minor, with additional populations along parts of the Atlantic coast in France and Ireland (Figure 2a). It is widely distributed across the Iberian Peninsula in the *Quercus ilex* L. and *Quercus suber* L. forests as part of their understory, from 0 to 1000 m above sea level [49]. Its round red fruits, measuring 1–2 cm, require a full year to mature and are harvested in late autumn and winter, coinciding with the flowering season [41].

*Prunus spinosa* L. (blackthorn or sloe) is a deciduous shrub reaching up to 5 m in height, characterized by its spiny branches (Figure 2b). It is widely distributed across Europe, Siberia, southwestern Asia, and northwestern Africa. In the Iberian Peninsula, it is commonly found along the forest margins and openings [69]. The small, globular drupes that reach roughly 1.5 cm in diameter ripen in late autumn and exhibit a characteristic blue–black coloration [69].

*Quercus* is a widespread genus of trees common across the northern hemisphere [70] (Figure 2c). Two species are commonly found and utilized in the Iberian Peninsula: *Q. ilex* (holm oak) and *Q. suber* (cork oak). *Quercus. ilex* is an evergreen tree native to the central–western Mediterranean. Its fruits are relatively small acorns, reaching up to 2 cm in length, which mature between November and January. Throughout the Iberian Peninsula, *Q. ilex* woodlands have traditionally been managed as open grazing areas, with large, isolated trees providing acorns for livestock grazing on the surrounding pastures [42,49]. *Quercus. suber* is also an evergreen tree native across the Mediterranean and along the Atlantic coast of the Iberian Peninsula that has long been used for cork production. Its acorns are bigger than those of *Q. ilex*, reaching 2–3 cm and maturing between September and January [45,49].

Although Mediterranean pines are often exploited for nuts, this project focused on the less-utilized green pine cones (Figure 2e) of three among the most common pine species in Catalonia: *Pinus halepensis* Mill. (Aleppo pine), *Pinus sylvestris* L. (Scots pine), and *Pinus nigra* Arnold (black pine) [43]. *Pinus halepensis* is a drought-tolerant species native to western Mediterranean coasts, occurring mainly at low altitudes [48]. It mainly flowers in spring and has a high production of cones [71,72]. On the contrary, *P. sylvestris* can be found across Eurasia, up to 2600 m [47]. It typically flowers between May and June, with the cones maturing for next two seasons [73]. *Pinus nigra* has a more fragmented distribution across Europe and W Asia, generally at altitudes from 800 to 1500 m [46], and it mainly flowers between March and May [74,75].

The genus *Rosa* is native across the Northern Hemisphere, from sea level to 1600 m in montane and Mediterranean regions [44,76,77] (Figure 2d). Representatives of the genus are robust, thorny shrubs that typically reach 1–3 m. Their dark red ovoid fruits develop from September to December [51,66,67,78]. The taxonomy of the genus remains unresolved due to the hybridization, high morphological variability, and subtle differences between species, which complicate their delimitation [76,79].

### 2.2. Literature Review of Traditional Uses

#### 2.2.1. Search Strategy

Although the project has a regional focus on Mediterranean forests, the search was conducted on a global scale to capture a broader range of traditional alimentary uses. This approach aimed to increase the diversity of traditional uses and provide better inspiration for potential innovative product development.

The objective of the review was to investigate the traditional uses of the five selected taxa (i.e., *A. unedo*, *P. spinosa*, *Quercus* spp., *Pinus* spp. and *Rosa* spp.; Figure 2), with a specific focus on the traditional alimentary uses of fruits and green cones. To capture a broader diversity of traditional uses, especially from regions where the target taxa do not occur, the search was conducted at the genus or family level at a global scale (i.e., *Arbutus* spp., *Prunus* spp., *Quercus* spp., *Rosa* spp., and family Pinaceae). Given the large number of cultivated species within the *Prunus* genus, the search focused primarily on traditional uses of *P. spinosa*, while also including 45 other wild shrub species of *Prunus*, selected from the Plants for a Future database [80] and complementary literature, with cultivated taxa excluded. For *Pinus*, given the low number of records retrieved when searching only by genus, and due to the minor structural differences among cones, the search was extended to encompass other representatives of the Pinaceae family.

We first carried out a comprehensive literature search in the English language across several online databases, including Scopus, Web of Science, SciFinder, PubMed, and Google Scholar. Searches were conducted individually for each taxon of interest. The most common combinations of keywords used were: *ethnobotany* AND *food* OR *edible* AND *[genus or species name]*, *[genus]* OR *[common name]* AND *fruit* AND *food* OR *edible* AND *use* OR *traditional* OR *knowledge*, *culinary* AND *[genus]*, *edible* AND *[genus]* AND *traditional*, *edible* AND *[genus]*, *[genus]* AND *traditional* AND *use* AND ethnobotan* AND nutrition*, *[genus]* OR *[common name]* AND ethnobotan* OR *“traditional knowledge”*. To capture additional traditional knowledge published in non-English sources, supplementary searches in Google Scholar were also performed in the French, Italian, Portuguese, and Spanish languages, using translated equivalents of the keyword combinations above. Further details on the search terms and strategies used for each genus or family, including variations in keywords across databases, are provided in the Supplementary Materials (Table S1). The searches were completed by the end of September 2024.

A complementary search of three renowned, open-access ethnobotanical databases (i.e., *Etnobotànica dels Països Catalans* (Ethnobotany of the Catalan Countries)) [81], Native American Ethnobotany [82], and Plants For A Future [80]) and Spanish ethnobotanical inventories [49,83,84,85,86,87,88] was also carried out. These searches did not employ specific keywords; instead, queries were generally performed at the genus level, and only alimentary uses were considered. The searches were completed by the end of October 2024.

#### 2.2.2. Reference Selection and Eligibility Criteria

The references obtained through the search were managed using Zotero software (version 7.0.27) [89], which facilitated the reference management and identification and removal of duplicates. All references were reviewed by two of the authors to minimize divergent interpretations of the eligibility criteria. Any disagreements were resolved through discussion and consensus with the remaining authors, who have extensive experience in ethnobotanical research. This process ensured a critical and consistent assessment of inclusion and exclusion criteria.

Titles and abstracts were screened first, and references showing potential ethnobotanical relevance were subjected to full-text review. References were included only when they met all of the following conditions: they contained a taxonomic name corresponding to one of the five target taxa at genus/family level and they explicitly documented a traditional alimentary use of the fruits or cones. Publications reporting mixed medicinal-alimentary uses were included only if the alimentary use was clearly and unambiguously stated. Conversely, publications were excluded when they did not mention traditional alimentary uses (e.g., those dealing exclusively with nutritional composition, toxicity, ecology, or other non-alimentary aspects) or when alimentary use was ambiguous. No restrictions were applied with respect to country of origin or publication date. References published in languages other than English, French, Italian, Portuguese, Spanish, or Catalan were excluded because they could not be reliably assessed by the review team. Included sources comprised peer-reviewed articles, reports, BSc, MSc and PhD theses, books, inventories, and database entries.

The complete list of references included in the study is provided in the Supplementary Materials (Table S2).

#### 2.2.3. Data Extraction

Data extracted from the selected publications were compiled into an Excel spreadsheet (Table S2). For each reference, the following information was recorded when available: (i) taxon and species, (ii) country, (iii) region(s), (iv) alimentary use, (v) additional observations regarding toxicity or precautions, and (vi) reference source (database or publication reference). If one reference contained several alimentary uses, they were entered in separate rows of the spreadsheet. Taxonomic nomenclature was harmonized using accepted plant names from Plants of the World Online (POWO) [90], and all synonyms were updated to their corresponding accepted names.

Alimentary uses were then recategorized into 16 alimentary categories: alcoholic beverage, non-alcoholic beverage, beverage (not specified), coffee substitute, baked goods, condiments, cooked, dried, flour, nutritional supplement, raw, sauces, savory preserves, sweet preserves, and sweets, with all remaining uses grouped under other uses. To visualize the distribution of the collected data, alluvial charts were made using RAWGraphs (version 2.0) [91].

### 2.3. Data Analysis of the Gathered Ethnobotanical Data

Two metrics were used for data analysis: use reports and distinct uses, enabling both quantitative and qualitative assessment of traditional knowledge. A use report was defined as each individual reference to the traditional use of a species per source or reference, providing a measure of documented knowledge in the consulted literature. One use report is equivalent to one row in the spreadsheet in Table S2. Recategorizing these use reports allowed identification of the diversity of applications for each taxon. A key limitation was the difficulty in quantifying traditional use reports, as many publications described multiple uses without specifying the number of informants citing each one. Therefore, in this study, each alimentary use citation per reference was counted as a single use report, which may introduce bias, as all sources were assigned equal weight.

A distinct use was defined as a unique use characterized by a specific purpose and preparation method, clearly differentiated from other reported uses. The primary outcome of the review was quantified through the number of use reports, while both metrics (use reports and distinct uses) are presented due to their complementary value.

We also used the 16 standardized alimentary categories to examine associations between alimentary uses and the studied taxa. However, the categories ‘Beverage (not specified)’ and ‘Other uses’ were excluded from the analysis, as they comprised heterogeneous applications that could not be meaningfully analyzed as single groups.

First, a Pearson’s chi-square test of independence was performed in R (version 4.2.1) using the chisq.test() function from the *stats* package [92] to assess the overall association between taxa and alimentary categories. Standardized residuals from the chi-square analysis were visualized for exploratory interpretation to identify the cells contributing most strongly to the global associations. The results were visualized using the function “corrplot” of *corrplot* package [93].

Due to uneven sampling among taxa and the presence of low expected frequencies, Fisher’s exact test was applied using the fisher.test() function in R with 100,000 Monte Carlo replicates to obtain an accurate *p*-value for the global associations. To identify specific taxon–alimentary category combinations that deviated significantly from expectations, separate Fisher’s exact tests were conducted for each cell, and the resulting *p*-values were adjusted for multiple comparisons using the Bonferroni method from the *stats* package [92].

## 3. Results and Discussion

### 3.1. Traditional Alimentary Uses Through Literature Search

The literature search yielded 2829 use reports from both publications and ethnobotanical databases. Of these, 1665 use reports were gathered from 335 publications, and 1164 from three ethnobotanical databases (Table S2). Over 50% of the use reports originated from the Mediterranean region, while the remaining 50% referred to other regions worldwide, predominantly other European countries and North America. Overall, the dataset incorporated information from more than 50 countries.

The majority of data entries related to the use of acorns from the *Quercus* genus (939 use reports), followed by *Rosa* (805 use reports) and *Prunus* fruits (634 use reports; Table 1). By contrast, taxa within the Pinaceae family were least represented, with only 147 use reports. Across all taxa, 54 distinct alimentary uses from 16 alimentary categories were identified. The greatest diversity was associated with the genera *Rosa* (36 uses), *Quercus* (35 uses), and *Prunus* (34 uses). In contrast, Pinaceae, although analyzed at the family rather than genus level, exhibited the lowest diversity (19 uses; Table 2). This could suggest that the consumption of green cones is relatively uncommon in alimentary contexts due to high content of resinous and phenolic compounds [64,94,95] that make the green cones difficult and unpleasant to eat unless correctly processed. While pine nuts are far more frequently used for food purposes [96], green cones are more commonly employed in medicinal applications, particularly for treating respiratory disorders [50,97].

**Figure 3:**
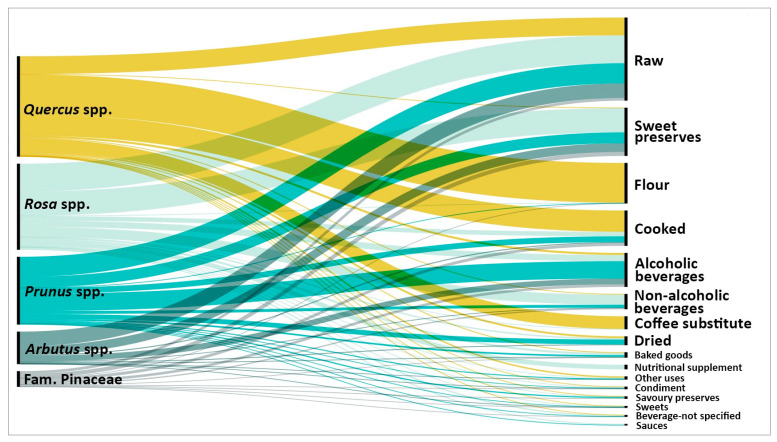
Alluvial diagram showing the distribution of alimentary categories (**right**) for each taxon (**left**). Flow width is proportional to frequency, illustrating the most common alimentary categories and the connections between taxa and categories.

The most frequently reported alimentary category was raw consumption (777 use reports, including those fruits exposed to post-frost softening, such as *P. spinosa* fruits), followed by sweet preserves (451), flour (379), cooked preparations (331), and alcoholic beverages (318), while the least frequent were different sauce preparations (15; Table 1, Figure 3).

**Figure 4:**
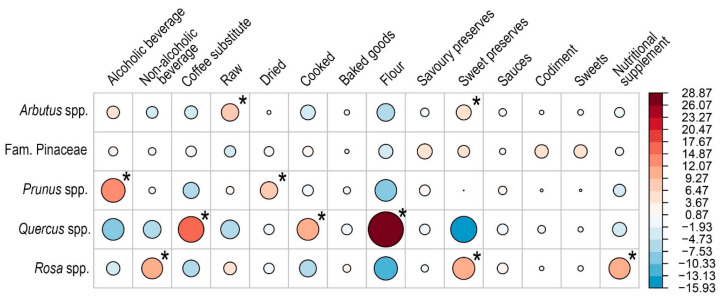
Adjusted Pearson’s chi-square residuals (represented by circle size and color shade) for fruits of each taxon across alimentary categories. Red indicates a positive association and blue a negative one. Asterisks denote significant positive associations of Fisher’s exact text (*p* < 0.001).

Differences in use report frequencies were found across taxa, with strong positive associations between certain taxa and specific use categories (Figure 4). Fisher’s exact test showed a significant association for the use of *Arbutus* fruits for raw consumption and preparation of sweet preserves, while their use was less pronounced, but still significantly associated with alcoholic beverages (Table S3, Figure 4). Use of green cones from the Pinaceae family was associated with production of savory preserves, while fruits of the *Prunus* genus showed a strong positive association with preparation of alcoholic beverages (mostly traditional liquors such as *pacharán/patxaran* and *ratafia/ratassia*) and dried fruit consumption (Table S3, Figure 4). Use of *Quercus* acorns was strongly associated with flour production, coffee substitutes, and a range of cooked preparations (Table S3, Figure 4). These uses reflect the high tannin content of *Quercus* acorns in their raw state, which requires processing methods such as leaching in water, roasting or boiling to reduce tannins and make the acorns more suitable for human consumption [60]. By contrast, fruits from the *Rosa* genus show strong association with use in non-alcoholic beverages, particularly herbal infusions and teas, and a lower but still significant association with raw consumption (Table S3). Due to their high vitamin C and E content [78], use of *Rosa* fruits is also positively associated with nutritional supplement use, particularly using the seeds (Table S3, Figure 4).

#### Overview of Traditional Uses by Taxa

- *Arbutus* spp.

In total, 304 food-related use reports for the genus *Arbutus* were identified. Traditional uses were dominated by raw consumption (137 use reports; 45%) and sweet preserves (81 use reports (27%), mainly jams and preserves), showing a strong association with *Arbutus* fruits (Figure 3 and Figure 4), likely due to their naturally high sugar content [57].

The third most frequent category was alcoholic beverages (54 use reports; 18%), which displayed considerable regional variation both in terminology (e.g., *rakija* [55], *koumaro* [98], *aguardente de medronho* [99]) and in preparation techniques. While most of the documented alcoholic beverages are produced by distilling fermented *Arbutus* fruits, we also found cases where the fruits were macerated in *grappa* or other high-grade alcohols [100,101,102], or where fermentation was stopped earlier and the fruits were used to make wine [103]. In some areas, otherwise discarded fruits were used for distillation [104], demonstrating potential for innovative products and supporting a zero-waste approach. Several companies in Italy, France, Spain, and Portugal already market *Arbutus* fruit–based products, such as jams, liqueurs, and sweets (e.g., Mestre Cacau, Portugal [105]; Sa Mariola, Italy [106]; Palacio del Deán, Spain [107]; Jean-Paul Vincensini et Fils, France [108]). Moreover, new alcoholic beverages are still being developed [31,109], highlighting the continuing commercial potential of these traditional products.

Less common uses included non-alcoholic beverages (e.g., cider, *vinetta* in Italy [99]), incorporation as condiments in yogurts and sweets, and, more rarely, dried fruits. Drying is uncommon because the fruit ferments rapidly [110], although steam-drying has been documented as a viable preservation method in traditional literature [111]. Due to their high sugar content and rapid fermentation, ripe specimens may naturally contain low levels of alcohol [104]. This should be considered when developing new products to avoid unwanted alcohol content.

Compared to other fruits, *Arbutus* fruits exhibited a relatively low diversity of uses, with 21 alimentary uses distributed across 10 categories (Table 2). No records were found for savory preserves, and only nine use reports for cooked dishes were recorded (Table S2). This limited use in savory contexts may be linked to the fruit’s pronounced sweetness, which makes incorporation into such preparations less suitable. Despite the relatively low diversity of traditional uses, it has been suggested that *Arbutus* fruits could be incorporated into a wider range of contemporary alimentary products, including condiments, yogurt preparations (either as pieces or flavorings), pie and pastry fillings, energy bars, and breakfast cereals [57,112,113,114]. This potential is supported by the fruit’s favorable nutritional composition, characterized by a high content of natural sugars and dietary fiber, notable levels of linolenic and malic acids, appreciable amounts of vitamins C and E (Table S4), and a substantial presence of anthocyanins, all of which indicate promising potential for their application in functional food products [41,56,57,115].

- *Prunus* spp.

A total of 634 use reports were gathered for the *Prunus* genus. The majority of uses involved consumption of raw fruits (188 use reports; 30%), though typically only when very ripe or post-frost. Fruits harvested in late summer or early autumn are often sour and bitter due to high polyphenolic content [116]; however, exposure to frost or traditional ripening techniques (e.g., storing in straw, in the shade, slightly buried, sun-drying until achieving a “chocolate-like” color) reduce this astringency [117]. Once fully ripe or frost-affected, the fruits develop a fine, slightly acidic, and palatable flesh, which is possible to consume raw [41].

The second most common use was the preparation of alcoholic beverages (163 use reports; 26%), including liqueurs (most notably *pacharán*, but also *arak* and *ratafia*) and wines, as was the case in Estonia [118]. There, the fruits were crushed and placed in a vessel with sugar and left to ferment until no further bubbling was observed in the water trap. The liquid was then poured into bottles to settle and later transferred to smaller bottles for consumption or storage [118]. For production of liqueurs via maceration such as *pacharán*, *P. spinosa* fruits are soaked in high-grade alcoholic drinks (e.g., *anís*, *orujo*) for six months [119]. Many companies already commercialize the *pacharán* in Spain [120,121], and to supply the fruit needed by companies, the cultivation of *P. spinosa* was established in Navarra, Spain in 1989. Along with fruit sourced from Eastern Europe, this region now represents an important source of *P. spinosa* fruits for industrial production [62].

Sweet preserves were the third most frequent preparation (101 use reports; 16%), dominated by jams and similar products (Table S2). Cooking reduces the fruit’s astringency, and the addition of sugar enhances palatability, which likely explains their widespread use in this category. Other notable uses included dried fruits (e.g., *lavashana* in Azerbaijan [62]), cooked fruits incorporated into soups or purees, and applications as condiments in both savory meat dishes and desserts (Table S2). Additional uses comprised non-alcoholic beverages (juices, infusions, lemonade, or *morse*, a traditional juice from Azerbaijan [122]), while unripe fruits were sometimes brined in a manner similar to olives [123].

While *pacharán* and jams are the most commonly commercialized products observed online (personal observation), a wide range of other products is also available on market, including aromas (Delsa, Spain [124]), frozen fruits (Faúndez Gourmet, Spain [125]), craft puree for flavoring beer (Amoretti, USA [126]), nectar (Blush & Berry, Bulgaria [127]), pickled fruits (Sams, Azerbaijan [128]) and even nutritional supplements (Vis Medicatrix Naturae, Italy [129]). This diversity of commercial applications reflects not only technological versatility but also the fruit’s intrinsic nutritional and functional value. Indeed, combined with its richness in vitamins B, C, and E (Table S4), anthocyanins, and other polyphenols [41,116], these market trends underscore its potential as a nutritious and functional ingredient. Moreover, the high anthocyanin content highlights additional opportunities for its use as a natural colorant and preservative in both food and pharmaceutical applications [13].

- *Quercus* spp.

The review focused on the *Quercus* genus, and a total of 939 food-related use reports were identified (Table 1). Alimentary use of acorns is well represented in traditional literature and over 35 distinct uses across 14 food categories were documented (Table 2).

Acorns have historically represented an important component of the diet of the native peoples of the Iberian Peninsula and are still used in traditional cuisine, particularly in western Spain [62]. The most frequently reported use was the preparation of acorn flour (372 reports; 40%), which served as the base for a wide variety of products, including sweet pastries (e.g., muffins, cakes, *coques*—traditional Catalan pastries), breads, and various savory foods such as porridge, crepes, omelets, and soups (Table S2). Acorn flour was also used as a thickener for soups and stews, providing both textural and nutritional value [61]. In North Africa, acorn flour was often mixed with wheat to produce traditional foods such as *kesra* bread and acorn-based couscous [130], while in Korea, it was used to prepare a tofu-like jelly called *dotorimuk*, utilizing the starch present in acorns [131]. This flour is rich in dietary fiber, vitamin A, and minerals (Table S4) and gluten-free, which makes it suitable for individuals with celiac disease [60].

A strong association was observed between the use of roasted acorns as a coffee substitute (119 use reports; 13%; Figure 4) and in a variety of cooked preparations (199 use reports; 21%). Most *Quercus* uses involved at least one form of heat processing (e.g., roasting or boiling) to reduce the bitter taste caused by tannins, which are abundantly found in acorns [80]. Once debittered, acorns can be dried, ground into flour, and used in porridges, breads, couscous, or other preparations (Table S2). Nevertheless, raw consumption was reported in 167 use reports (18%), particularly when acorns contained lower tannin levels and were naturally sweet [132]. Tannin concentrations have been shown to vary both among and within *Quercus* species [60], which is also reflected in traditional literature [132]. Other applications include alcoholic beverages (liqueurs), incorporation into sweets (e.g., *turrons/torrons*), and non-alcoholic drinks, such as *horchata/orxata* (Table S2). Eight use reports documented the use of acorn oil, which possesses nutritional properties comparable to olive oil [60].

The study by Zocchi et al. [133] showed that acorns were historically used primarily in times of famine, and their use has declined in recent times. However, interest in acorns is now resurging due to their rich nutritional properties. Several innovative products have already been developed [134,135,136,137,138,139,140], and research indicates that acorn flour production has notable economic potential [141]. This renewed interest is also evident in the growing variety of acorn-based commercial products currently available on the market: acorn flour is sold directly (De bellota, Spain [142]) or incorporated in baked goods (bread from Herdade do Freixo do Meio, Portugal [143], breadsticks from Abuela Paula, Spain [144], and cookies from Marcie Mayer Maker Lab, Greece [145], etc.), while whole acorns or other acorn-derived preparations are employed in products such as alcoholic beverages (La Extremeña, Spain [146]), juices (Rural Farmer, Republic of Korea [147]), vegan burgers (Herdade do Freixo do Meio, Portugal [148]), and coffee (Dary Natury, Poland [149]).

- Pinaceae Family

Targeted searches on the traditional alimentary uses of cones from the *Pinus* genus yielded limited references; therefore, the search was broadened to include other genera within the Pinaceae family (i.e., *Abies*, *Cedrus*, *Tsuga*, *Pseudotsuga*, *Larix*, *Picea*). A total of 147 food-related use reports were collected for green cones (Table 1). The most cited genus was *Pinus* (88 use reports), followed by *Picea* and *Abies* (42 and 14 use reports, respectively, Table S2).

Literature on the edible use of green cones is scarce, with more information available on pine nuts, young shoots, leaves, and resin. This scarcity is reflected in our results: only 19 distinct alimentary uses were documented, distributed across 11 food categories (Table 2). The most common uses of green cones were sweet preserves (38 use reports; 26%), including syrups, jams, and preserves, followed by cooked preparations (27 use reports; 18%), mostly involving roasting the green cones (Table S2). Raw consumption was reported in 25 use reports (17%), particularly when cones were very young [150]. Preparation of alcoholic beverages was also frequent (24 use reports; 16%), often as an aromatic addition to liquors such as *ratafia* and *grappa* [97]. However, in Romania, young shoots and cones of *Abies alba* Mill. are distilled to produce brandy called *pálinka* [151]. Non-alcoholic beverages, primarily teas and infusions, were also prepared, especially from *Picea* and *Pinus* species (Table S2). Other uses included savory preserves (7 use reports; 5%), such as brined or pickled cones, and condiments (6 use reports; 4%), including smoking meat or fish using green cones and dried and ground green cones for seasoning [118]. Despite the limited references in traditional use, green pine cones show potential for use as natural stabilizers in the food industry due to a rich polyphenol and terpenoid profile that provides strong antioxidant, anti-aging, and radical-scavenging properties [64].

In Spain, two companies market cone-syrup-based products (Sudàvel [152] and JoannArteida [153]). These are primarily sweet syrups, confections, vinaigrettes, liqueurs, and cone nectar. Notably, renowned chefs such as Ferran Adrià at El Bulli (Catalonia, Spain) have incorporated various traditional uses of *Pinus* spp. into haute cuisine, including cold-infused pine oils, aromatic teas and syrups, and pine-flavored desserts such as granitas, meringues, and dehydrated gelatine, as well as jams, syrups, and pickles, demonstrating the considerable potential of such products [154]. Importantly, green cones are often generated as by-products of spring forest management [63], and their utilization could provide additional economic benefits from material that would otherwise be discarded.

- *Rosa* spp.

The search encompassed the entire *Rosa* genus, and a total of 805 use reports were collected for *Rosa* fruits (Table 1). Documentation of traditional uses of *Rosa* fruits is extensive, and we recorded more than 36 alimentary applications distributed across all 16 alimentary categories (Table 2, Figure 3).

The most frequently reported use was raw consumption of ripe fruits (260 use reports; 32%; Table S2, Figure 3), followed by sweet preserves (227 use reports; 28%), such as jams, syrups, jellies, and compotes. The third most common category was non-alcoholic beverages (94 use reports; 12%), most often prepared as teas, herbal infusions, or fruit juices (e.g., *kissel* in Estonia [54]). Some of these products are already commercially available [155,156].

Alcoholic beverages (58 use reports; 7%) were also documented, ranging from macerating fruits in brandies and liqueurs [97,157] to using the fruit to flavor wine and beer [51,158]. A use recorded exclusively for *Rosa* fruits was their consumption as a nutritional supplement (43 use reports, Figure 3), where ground seeds rich in vitamin E are incorporated into other foods [80]. *Rosa* fruits are also notable for their high vitamin C content, with fresh fruits containing, on average, around 400 mg/100 g (Table S4) [51,53,66,67,78]. Interestingly, despite this remarkable concentration, only 11 use reports specifically mentioned vitamin C. Additionally, the fruits are a source of vitamins A, B, and E, and rich in fiber, calcium, magnesium, and potassium (Table S4) [66,68,115,159]. Information was also collected on preservation methods, including freezing, drying (either in the sun or oven), and pasteurization of juices. To retain vitamin C, fruits are often harvested before frost and briefly heat-treated after being frozen [160]. While no scientific studies were found replicating vitamin C conservation in such conditions, studies indicate that over 70% of vitamin C can be retained in *Rosa* fruits stored at −18 °C for more than 600 days [161].

Less common applications include the use of fruits in soups, powdered fruit as a flour substitute, or incorporation into pastries. *Rosa* fruits may also be dried and stored for later use, while roasted and ground seeds can serve as substitutes for coffee or cacao [162]. Additional uses include preparation of ketchup, chutneys and sauces, ice cream, *pemmican*, roasted snacks, and homemade vinegars (Table S2). Some of these uses have already been commercialized internationally (yogurt from Andechser Natur, Germany [163], *grappa* from Il Buongustaio, Italy [164], sweets from Sweets for Health, Russia [165], sweet preserves [166,167], and powders marketed as nutritional supplements [168]). Moreover, its fruits have appeared in contemporary gastronomy, including fine-dining contexts such as NOMA [20]. Their antioxidant and bioactive compounds, including ascorbic acid and β-carotene, also show potential for use in commercial products to delay rancidity in fatty materials during food manufacturing, to promote human health [78] and as food colorant [169].

### 3.2. Potential Adverse Effects

Out of a total of 2829 use reports, 434 (15%) included warnings or precautionary notes related to alimentary use in four of the five target taxa (Table 3). These reports primarily addressed potential adverse effects and preparation methods to mitigate risks. Warnings were most frequently associated with use of *Quercus* spp. acorns, followed by *Rosa* spp., *Prunus* spp., and *Arbutus* spp. fruits, with 195, 143, 88, and 7 use reports, respectively.

Among the taxa, the most serious adverse effects concerned the consumption of *Prunus* spp. seeds. Warnings against seed ingestion were documented in 88 use reports, representing 14% of all *Prunus* alimentary use reports (Table 3). These cautions emphasized avoiding seed consumption, particularly when very bitter, as bitterness is often associated with the presence of toxic compounds. Seeds of *Prunus* species contain cyanogenic glycosides, such as amygdalin and prunasin, which have toxic and potentially carcinogenic effects [13,170]. According to EFSA, ingestion of as few as three small apricot (*Prunus armeniaca* L.) kernels may be sufficient to reach the acute reference dose for amygdalin in adults [171,172,173]. In wild *Prunus* species, including *P. spinosa*, seeds are generally not intentionally consumed, although accidental ingestion may occur. Intact seeds typically pass through the digestive system without releasing cyanogenic glycosides, which are liberated only when the seed coat is damaged [172]. Notably, one traditional source reported that flour produced from *Prunus ilicifolia* (Nutt. ex Hook. & Arn.) Walp. seeds becomes safe for consumption after leaching and can be used in bread, soups, and *atole*, a traditional Mexican hot beverage [123]. Because amygdalin is water-soluble [174,175], leaching crushed seeds may reduce cyanogenic glycoside concentrations to safe levels. Rodríguez-Blázquez et al. [176] reported low amygdalin concentrations in *P. spinosa* seeds; however, further research is required to define safety thresholds and limitations for such use of *P. spinosa*. Aside from seed-related toxicity, no other adverse effects were reported in traditional sources, suggesting that *Prunus* fruits themselves are generally safe for use in the food industry.

Following *Prunus*, the second most serious adverse effects were associated with *Quercus* spp. acorns, for which 195 use reports emphasized the importance of proper processing to reduce tannin content (Table 3). One report also noted that excessive consumption could cause headaches [177], potentially related to high tannin intake. Tannins are polyphenolic compounds responsible for acorn bitterness that reduce nutritional quality of food by forming complexes with proteins, polysaccharides, digestive enzymes, and metal ions, thereby limiting nutrient digestibility and absorption. When consumed in large quantities, tannins are considered anti-nutritional and undesirable [178,179,180]. Their concentration in acorns varies widely with species, season, fruit maturity, and growing conditions [60,180,181], ranging from less than 1% to more than 10% of dry weight [179,180,182]. Recommended daily doses for animals range from 317 mg/kg for cats to 3000 mg/kg for ornamental fish [183], while for humans, the daily intake limit is 560 mg/kg [180]. To mitigate these risks, numerous traditional processing methods have been developed to lower tannin concentrations in acorns. Because tannins are water-soluble, they can be removed by repeated soaking in cold or hot water or by immersion in running streams. Hot-water treatments reported faster tannin removal [184], but cold-water leaching may result in higher-quality flour [185]. Other traditional techniques include burying acorns in moist soil over winter, soaking them in lye prepared from hardwood ash, or allowing germination, after which acorns may be consumed raw or incorporated into prepared dishes [61]. Many modern studies have replicated these approaches and demonstrated successful tannin reduction [135,179,180,186,187,188].

Less severe but still relevant adverse effects were recorded for other taxa, having important considerations for food development. In references for *Arbutus* spp. fruits, six use reports warned of the potential presence of alcohol (Table 3), advising moderation due to possible intoxication [104]. One reference also reported that overconsumption could cause headaches [189], highlighting the need to prevent unwanted fermentation when developing innovative products.

In *Rosa* spp. fruits, 144 use reports cautioned against irritating hairs surrounding the seeds inside the fruit (Table 3), which can cause skin reactions upon direct contact [190]. Prior to consumption, these hairs must be carefully removed and the fruit thoroughly washed, as ingestion may cause irritation of the mouth, throat, and digestive tract, as well as discomfort during defecation [81]. Some modern studies and patents describe sieving techniques to remove these hairs [191,192], although few studies were found.

For the uses of green pine cones, no adverse effects were recorded, aside from a single source recommending blanching to reduce astringency [185], suggesting a low risk of negative effects when consuming young pine cones.

No references regarding potential allergenic effects were recorded in traditional literature. However, modern studies indicate that allergic reactions are possible, particularly for acorns [193,194,195] and *Rosa* spp. [196].

Consistent with previous studies, our findings confirm that traditional processing methods to mitigate potential risks are extensive and have been successfully integrated into modern food practices. This empirical knowledge, refined over generations, remains crucial for understanding both the potential adverse effects of WEPs and the preparation techniques needed to ensure their safe consumption [27,28,195]. Accordingly, documenting not only uses but also associated warnings is essential, as these reflect time-tested strategies for minimizing harmful effects. Nevertheless, modern analytical techniques capable of identifying toxic compounds (e.g., tannins and cyanogenic glycosides), alongside expert evaluations by international bodies such as the European Food Safety Authority (EFSA), remain critical for confirming food safety [115,197].

### 3.3. From Traditional Knowledge to Innovative Food Products: Opportunities and Limitations

The review of traditional knowledge provided valuable insights for the development of innovative alimentary products based on WEPs. More than fifty distinct uses of fruits and green cones across the selected taxa were identified, highlighting their broad culinary potential (Table 2). However, the most frequently reported uses, primarily raw consumption, preservation in sweet preserves, and alcoholic beverages (Table 1, Figure 3), may not always be optimal for developing healthy, nutritional, and functional foods, which are currently increasing in demand [198].

This review also identified several underutilized traditional practices with strong potential for contemporary food innovation. These include fermented and other non-alcoholic beverages, dried fruits used in *pemmican*, fruits preserved in brine, and applications in sauces and condiments. Although these uses appear in less than 1% of all reported use records, their exploration is essential for supporting research and development of novel food products. Their preservation has been made possible largely through extensive ethnobotanical studies [20,199], yet these practices remain at risk of disappearing, making their documentation and revival all the more urgent.

Beyond offering inspiration for product diversification, traditional knowledge also provides critical information on the safe use of WEPs, including awareness of potential adverse effects and the processing steps required to ensure safe consumption [27,28,200], as discussed in Section 3.2. Potential adverse effects.

Traditional practices further offer detailed guidance on preparation and preservation methods that help maintain nutritional quality, an important consideration given the sensitivity of vitamins, minerals, and antioxidants to processing conditions [201,202]. For four of the five taxa reviewed (*A. unedo*, *P. spinosa*, *Quercus* spp. and *Rosa* spp.), substantial data on nutritional composition is already available (Table S4), highlighting the importance of appropriate processing strategies. Processing parameters such as method, duration, and temperature play a decisive role in nutrient retention [159,201,203], as unsuitable conditions can result in significant losses of vitamin C [203,204] and other bioactive constituents [202]. Nevertheless, several techniques have demonstrated the capacity to preserve beneficial compounds while maintaining sensory quality [205], including freeze-drying [113,114,206], infrared freeze-drying [207], freezing [116,208], cutting fruits prior to drying [203], and selected cooking methods [201,202]. The incorporation of these fruits into various food products (e.g., *A. unedo* fruits in kefir [113], yogurt [114], spirit drinks [31], and bread [209]; *P. spinosa* fruits in marmalade [202] ice cream [210], and kombucha [211]; *Quercus* spp. acorns in biscuits [212] and bread [137]; *Rosa* spp. fruits in ice cream [169] and turkey burgers [213]) further supports their functional potential, enhancing both nutritional value and sensory characteristics. Collectively, these findings demonstrate that the development of functional foods enriched with WEP-derived bioactive compounds is both feasible and well supported by existing scientific evidence.

At the same time, the high abundance of secondary metabolites in these species can negatively affect organoleptic properties and, in some cases, nutritional quality. For example, after eight months of frozen storage, fresh juice from *Rosa roxburghii* Tratt. fruits exhibited a shift in aroma from fruity to unpleasant [214]. Another example is *P. spinosa* fruits, where high tannin levels cause pronounced astringency, which limits their food applications [59]. Traditional sources frequently recommend consuming *P. spinosa* fruits only after exposure to natural frost, a process associated with reduced tannin concentrations [81,215]. Post-harvest freezing for several weeks could mimic this effect, although no studies have yet scientifically evaluated this approach, requiring scientific validation. Similarly, the high tannin content of *Q. ilex* acorns represents a major limitation, as tannins confer astringency and impair digestion, thus reducing nutrient absorption [61,188]. Green pine cones present additional challenges due to their richness in phenolic and terpenoid compounds, which contribute to a bitter taste [63] and complicate dosage determination, thereby requiring creative culinary approaches. Product standardization is further hindered by substantial variability in volatile secondary compounds, which depends on species, geographic origin, and environmental conditions at harvest [64,65,216]. This natural variability complicates efforts to achieve consistent flavor profiles and uniform product quality.

Scaling traditional preparations to industrial levels presents further challenges. Traditional methods designed for household-scale production are not easily transferable, and the lack of suitable machinery and processing facilities increases complexity and cost [61]. Industrial production requires species-specific equipment to reduce labor, minimize costs, and ensure profitability, but these investments have high initial costs.

Finally, pronounced seasonality, strong inter-annual and spatial variability in production [217] can increase the logistical complexity and cost of harvest and processing. Diversification of harvested species has been proposed to buffer against these limitations [218].

### 3.4. Legal Considerations for the Utilization of Wild Edible Plants

An essential aspect of using WEPs in contemporary food systems is ensuring full compliance with the European Union’s food-law framework regulating their commercialization. Before any product can be placed on the market, the legal status of each taxon and of the specific plant parts intended for use must be verified according to current EU food legislation. This verification is carried out by consulting the competent national food-safety authority or the EU Novel Food Status Catalogue [219], which indicates whether a species and plant parts are already authorized and which uses are permitted.

For taxa and plant parts that are not yet authorized, products with documented traditional food use prior to 15 May 1997 are not considered “novel” and therefore are not subject to the Novel Food pre-market authorization procedure established by Regulation (EU) 2015/2283 [220]. In such cases, evidence demonstrating significant consumption within the Union before that date must be compiled and submitted to the competent national food-safety authority, which evaluates the documentation and initiates the corresponding authorization process. When no adequate evidence exists, the product is considered a Novel Food and must undergo the full pre-market authorization procedure under Regulation (EU) 2015/2283 [220]. Depending on the outcome of this assessment, the plant species and their parts may be authorized for use or remain non-authorized, in which case commercialization is prohibited.

Among the taxa considered here, fruits of *A. unedo*, *P. spinosa*, *Q. ilex*, *R. canina*, *R. rubiginosa*, as well as the cone syrup of *P. sylvestris*, are already authorized at the European level for specific uses [219], in many cases thanks to the substantial body of traditional knowledge that has been preserved for thousands of years and systematically documented. In contrast, cones of *P. halepensis* and *P. nigra*, the fruits of *Q. suber*, and the remaining *Rosa* species are currently under evaluation by the Spanish food-safety authority (AESAN) based on submitted evidence of traditional food use.

In addition to the ingredients, consideration must be given to food additives and allergen declaration. Any additives used must comply with Regulation (EC) 1333/2008 [221] and be applied at authorized doses with homogeneous distribution within the product. Labeling must follow Regulation (EU) 1169/2011 [222], which includes a legally defined list of allergens that must be declared. All allergens present must be clearly highlighted, and although the fruits considered in this project (*A. unedo*, *P. spinosa, Quercus* spp., *Pinus* spp., *Rosa* spp.) do not have specific legal restrictions as allergens, potential cross-contamination must be indicated if relevant. Attention to these aspects ensures both legal compliance and consumer safety while preserving the traditional character of the products.

Another consideration is compliance with the legal framework regulating the collection of WEPs, which varies between countries. In Spain, these activities are regulated to ensure sustainability, safety, and compliance with national [223] and regional legislation [224,225]. Regulations require landowners’ authorization, adherence to management plans, and, in the case of commercial operations, formal contracts.

Good collection practices that include avoiding contaminated areas, industrial zones, mines, roads with heavy traffic, and areas frequented by animals must also be followed. Collectors must comply with labor standards and adopt sustainable harvesting practices to avoid overexploitation of natural resources [226,227,228], while ensuring a sufficient quantity of fruits and cones remains available for wildlife consumption and natural regeneration. Although information on natural yield rates of Mediterranean WEPs is scarce, available data suggest that some traditionally consumed species are abundant and productive, making sustainable collection for product development a viable option [16].

## 4. Conclusions

There is an urgent need to identify new sources of food to support global food security and enhance dietary diversity. Traditionally used WEPs represent a promising resource, as their long-standing uses and processing practices provide a foundation for the development of safe, innovative, and functional food products. This study analyzed the traditional alimentary uses of five taxa (*A. unedo*, *P. spinosa*, *Quercus* spp., *Pinus* spp. and *Rosa* spp.), revealing a wide diversity of preparations and statistically significant associations between specific fruits and culinary applications.

The results highlight the role of traditional knowledge not only in identifying edible species but also in guiding appropriate processing methods and safety precautions. While these practices provide a useful basis for innovation, their adoption in modern food systems will require targeted technological solutions to ensure product safety, nutritional quality, and scalability.

The successful integration of WEPs into contemporary food systems depends on coordinated efforts across research, production, land management, and policy. Systematic documentation of ethnobotanical evidence of historical consumption is essential to support regulatory approval and demonstrate long-standing safe use within the European framework, while reviews, such as this play a complementary role by organizing and making these data more accessible to researchers.

Food producers and small-scale enterprises play an important role by developing functional and value-added products based on WEPs, supported by local processing facilities and collaborative value chains involving forest owners, processors, and local communities. Integrating WEP harvesting into forest management plans can further promote multifunctional forest use, contributing to biodiversity conservation, fire prevention, and rural development.

Finally, supportive policies and regulatory frameworks are necessary to encourage wider adoption of WEPs. Recognition of traditional consumption, along with education, training, and cross-sector collaboration aligned with circular economy principles, can support the responsible use of native fruits.

By combining traditional knowledge with scientific research, food development, forest management, and regulatory compliance, there is substantial potential to develop innovative, safe, and environmentally responsible food products that enrich diets while preserving both cultural and ecological heritage and will be key to unlocking the full potential of native WEPs in modern food systems.

## Figures and Tables

**Table 1:** Number of use reports for each alimentary category across five selected taxa and their total values obtained in the review.

	*Arbutus* spp.	Fam. Pinaceae	*Prunus* spp.	*Quercus* spp.	*Rosa* spp.	TOTAL
Raw	137	25	188	167	260	777
Sweet preserves	81	38	101	4	227	451
Flour	0	1	2	372	4	379
Cooked	9	27	54	199	42	331
Coffee substitute	0	0	0	119	3	122
Dried	8	0	45	17	13	83
Baked goods	7	2	15	8	19	51
Nutritional supplement	0	0	0	0	43	43
Condiment	2	6	6	5	6	25
Savory preserves	0	7	11	2	4	24
Sweets	1	5	4	5	4	19
Sauces	0	0	6	0	9	15
Other uses	1	0	9	13	7	30
Alcoholic beverage	54	24	163	19	58	318
Non-alcoholic beverage	4	11	27	7	94	143
Beverage—not specified	0	1	3	2	12	18
TOTAL	304	147	634	939	805	

**Table 2:** Number of use reports, alimentary uses and categories across five selected taxa.

	Use Reports	Number of Alimentary Uses [Out of 54]	Number of Categories [Out of 16]
*Arbutus* spp.	304	21	10
Fam. Pinaceae	147	19	11
*Prunus* spp.	634	34	14
*Quercus* spp.	939	35	14
*Rosa* spp.	805	36	16

**Table 3:** Number of use reports (and percentages of total number per taxa in brackets) that cited possible adverse effects and recommendations of the consumption fruits or green cones of the five target taxa.

	Plant Part	*Arbutus* spp.	Fam. Pinaceae	*Prunus* spp.	*Quercus* spp.	*Rosa* spp.
Adverse effects						
Overconsumption may cause headaches	F	1 (<1%)			1 (<1%)	
Possible alcohol content—risk of intoxication	F	6 (2%)				
Avoid seed consumption—toxic compounds	Se			88 (14%)		
Presence of tannins	F				194 (21%)	
Presence of irritant hairs	F/Se					138 (17%)
Consume in moderation—it causes constipation	F					6 (1%)
TOTAL		7 (2%)	NA	88 (14%)	195 (21%)	144 (18%)

F—fruit; Se—seed.

## Data Availability

No new data were created or analyzed in this study. Data sharing is not applicable to this article.

## Supplementary Materials

The following supporting information can be downloaded at: https://www.mdpi.com/article/10.3390/foods15010150/s1, Table S1: The details regarding the specific search strategies: the search terms, keywords and search engines employed for each genus or family. Table S2: Results for the literature search for each taxa consulted and the list of references included. Table S3: Results of Fisher’s exact test. with 100,000 Monte Carlo replicates. The resulting *p*-values were adjusted for multiple testing using the Bonferroni method. Table S4: Data are expressed per 100 g of fresh weight. Dietary Reference Values (DRVs) correspond to those defined in the Annex to Directive 90/496/EEC or to EFSA reference intakes for healthy adults. Values highlighted in bold can be classified as “source of” as defined in the Annex to Directive 90/496/EEC or as amounts provided for by derogations granted according to Article 6 of Regulation (EC) No. 1925/2006 of the European Parliament and of the Council of 20 December 2006 on the addition of vitamins and minerals and of certain other substances to foods. Results originally presented on a dry-weight basis were converted to fresh weight using fruit-specific moisture content. Observed values may vary due to analytical limitations and biological factors. The literature search was conducted at the species level for *Arbutus unedo*, *Quercus ilex*, *Prunus spinosa* and *Rosa canina*. No scientific literature reporting comparable nutritional data was found for *pinus* spp.
